# An Edge Computing Based Smart Healthcare Framework for Resource Management

**DOI:** 10.3390/s18124307

**Published:** 2018-12-06

**Authors:** Soraia Oueida, Yehia Kotb, Moayad Aloqaily, Yaser Jararweh, Thar Baker

**Affiliations:** 1 Politehnica University of Bucharest, 060042 Bucharest, Romania; Soraia.Oueida@upb.ro; 2 American University of the Middle East, Eqaila 250 St, Kuwait; Yehia.Kotb@aum.edu.kw; 3 Gnowit Inc., 7 Bayview Road, Ottawa, ON K1Y3B5, Canada; 4 Jordan University of Science and Technology, Irbid 22110, Jordan; YiJararweh@just.edu.jo; 5 Liverpool John Moores University, Liverpool L3 3AF, UK; T.Baker@ljmu.ac.uk

**Keywords:** edge computing, cloud computing, smart city, smart healthcare management, emergency department, Petri net workflow, workflow soundness

## Abstract

The revolution in information technologies, and the spread of the Internet of Things (IoT) and smart city industrial systems, have fostered widespread use of smart systems. As a complex, 24/7 service, healthcare requires efficient and reliable follow-up on daily operations, service and resources. Cloud and edge computing are essential for smart and efficient healthcare systems in smart cities. Emergency departments (ED) are real-time systems with complex dynamic behavior, and they require tailored techniques to model, simulate and optimize system resources and service flow. ED issues are mainly due to resource shortage and resource assignment efficiency. In this paper, we propose a resource preservation net (RPN) framework using Petri net, integrated with custom cloud and edge computing suitable for ED systems. The proposed framework is designed to model non-consumable resources and is theoretically described and validated. RPN is applicable to a real-life scenario where key performance indicators such as patient length of stay (LoS), resource utilization rate and average patient waiting time are modeled and optimized. As the system must be reliable, efficient and secure, the use of cloud and edge computing is critical. The proposed framework is simulated, which highlights significant improvements in LoS, resource utilization and patient waiting time.

## 1. Introduction

Smart healthcare is one of the major components of smart cities. The field of smart healthcare emerges from the need to improve the management of healthcare sector, better utilize its resources, and reduce its cost while maintaining or even enhancing its quality level. Resources in the healthcare sector can broadly be classified to consumable resources and non-consumable resources. Consumable resources include those resources that they decay and expire by time like all medical aids and tools. Non-consumable resources, on the other hand, are those resources that do not expire by time. Among the non-consumable resources are the human resources such as physicians, nurses, registered nurses and all the human capital involved in the process of healthcare. The human capital of the healthcare sector is a very expensive resource and utilizing this resource in an efficient way is a step ahead towards a complete smart healthcare system.

During the last decade, the exponential increase in adults suffering from chronic diseases dramatically increased the need to find innovative approaches to handle the process of healthcare in a way to be smart with respect to service efficiency and cost [1]. The nature of the healthcare sector requires a reliable and efficient way to deal with the huge amount of data all the time. Thanks to cloud and edge computing, designing and developing smart healthcare is now possible since it has been proven that cloud is more reliable than regular servers. In addition, the privacy of patients is still secured by cloud computing providers [2]. The opportunities and challenges of adopting new technologies are evaluated in [3], and the benefits of migration from traditional healthcare central systems into distributed cloud-fog-based healthcare systems, and the corresponding need for resource allocation, direction and strategy, are also discussed. Moreover, with the support of mobile cloud computing, wireless body area networks can be enhanced for deployment of healthcare applications [4]. Cloud and edge computing provide a secure, safe, platform for smart healthcare. In a later research, Tuan et al. [5] highlighted and demonstrated the importance of the Internet of Things (IoT) and fog computing to provide better reliable health services.

Smart healthcare research can broadly be classified into two main categories: patient related category and process related category. Patient related category includes but is not limited to research that targets wearable devices for collecting data about patients to be reported to medical institutions. Examples of research in this category is the work done by Esposito et al. [6]. In their work, they develop a smart mobile self-configuring architecture that allows rapid personal health monitoring using the recent developments in sensors and mobile embedded devices. The authors in [7] discuss the importance of wearable devices and applications to collect data about status of patients. This of course is supported by edge, cloud computing and IoT [8]. Shah et al. prove the importance of IoT and smart devices in improving healthcare services [9]. Moreover, Sodhro et al. present in their research another example under this category. They develop a single chip based wireless monitoring system to collect real-time ECG (ECG stands for Electrocardiography) data and monitor human heart activities [10].

The second category is the process related research. In this category, research is concerned with the enhancement of policies to ensure many aspects in the healthcare sector. Among those aspects are resource utilization, resource scheduling, quality of service and many other aspects related to process definition and management. Examples of research in this category is the work done by Hossain et al. In their work, they study the importance of IoT in telemedicine and propose a model for an IoT-based health prescription assistant. The model helps patients to properly follow doctors’ recommendations [11].

Industrial processes compete for resources [12] while trying to maintain a required robust quality level of provided services [13]. Healthcare systems are dynamic and complex systems that have unpredictable behavior and that is why healthcare systems need to manage their resources and services efficiently. As mentioned earlier, we classify resources in two main categories: consumable and non-consumable. Consumable resources are those that expire through use, including beds, sheets, chairs and any other items that cannot be shared or have an expiry date, while non-consumable resources, such as human labor, do not expire. This research focuses on the process level and particularly on modeling the process of resource assignment in an emergency department. To achieve this, we use Petri nets as a framework to model our processes and protocols. Controlling the flow process in an emergency department is not trivial since the system dynamic behavior is complex. Kotb et al. present a control flow process to maintain synchronization among activities using Petri nets and applied their proposed framework on healthcare sector [14]. Petri nets are graphically represented mathematical frameworks to represent systems that are concurrent.

Emergency Departments (EDs) are modeled using Petri nets. One of the quality factors in EDs is the length of stay (LoS), which, when it rises, it brings the average waiting time of patients up. This causes overcrowding, which, in turn, leads to patient dissatisfaction [14]. Increasing the capacity of EDs and adding extra resources is a possible solution, but these options are not always available due to budget constraints and the high costs of providing extra facilities [15]. This motivates the authors of this paper to target the problem of resource assignment and process modeling and management of an ED using Petri nets. In this process, a new Petri net subclass is proposed called Resource Preservation Net (RPN). We apply our proposed framework in an ED located in Lebanon. This study uses simulation to prove the feasibility of the proposed framework to reduce waiting time and patient LoS during visits to the ED with the help of cloud-fog technology. This alleviates overcrowding and reduces the waste of resources [16]. Internal and external ED processes are simulated, including registration, triage, examination, radiology and billing. The chosen ED is the emergency department of a hospital in Lebanon that has been recognized since 1952. This ED consists of two buildings connected by an underground tunnel, and each building has a separate emergency room (called herein as ER A and ER B). The ED serves more than 40,000 patients annually, and is open 24/7. The RPN by itself is a mathematical-based framework that we use to model the problem of non-consumable resource assignment. It is a part of a bigger project that targets the optimization of resource scheduling and assignment. The RPN is meant to model the problem of non-consumable resources and assign them when required. The main contribution of this subclass of Petri net is to ensure that non-consumable resources are always preserved, all siphons are controlled, and the system is safe and sound. The soundness of the RPN is supported in this paper with a few defined Theorems. The soundness of the RPN is not only dependent on the structure of the Petri net and its initial marking but also on the capabilities required by the undertaking task and the resource capabilities available. If the designer follows the constraints defined by the Theorems proposed in this paper, the resultant system is guaranteed to be safe and sound. In this paper, the RPN is integrated with edge computing. Every resource has their own smart device that communicates with the cloud to inform the cloud with the status of that resource. When the resource is assigned, the cloud sends the assignment request to the edge and, when the resource becomes idle, the edge informs the cloud for the following assignment. This paper integrates a process modeling methodology that is the RPN with the edge computing framework for better performance and higher reliability.

This paper is organized as follows: Section 4.1 demonstrates the preliminaries related to this paper. Section 2 presents the background of Petri nets and related work. Section 3 discusses the proposed RPN framework. Section 4 presents the mathematical model and system validation. Section 5 presents the relationship between IoT and healthcare and validates the proposed model. Section 6 demonstrates the generality of the proposed framework by applying it to robotics. Section 7 shows the RPN applications to smart healthcare, which is the main focus of this paper. The simulation results are provided in Section 8. Finally, Section 9 presents the conclusion and proposes future work.

## 2. Related Work

The importance of deploying IoT of mobile nodes into healthcare (i.e., medical team and stuff) is adopted in order to improve the system and thus leads to a better allocation of resources and an enhancement in performance measures. Healthcare is always considered as a very complex system and with the increase in population and patient surge, the medical care demand is increasing and thus the importance of transforming the healthcare system into a smart healthcare to coop with nowadays technological aspects. Currently, the healthcare sector is facing a great number of problems due to exponential increases in population and chronic diseases. As traditional health technology cannot solve these challenges, cloud computing was introduced to address the problems of data and resource utilization. Cloud computing is a recent technology in the IoT era, and it is the best approach to enhance medical services due to its multi-tenancy, flexibility and remote delivery [17,18]. A 2016 study by Dubey and Vishwakarma highlights the application of cloud computing in healthcare, and the key principles required to build a smart healthcare system in a smart city. This paper also discusses the common limitations and problems faced when adopting cloud computing for healthcare [19]. Mobile cloud computing and wireless body area networks can also be enhanced by the deployment of smart healthcare applications [4]. A study by Wan et al. highlighted the methodologies required to transmit data to a cloud, perform cloud resource allocation and apply data security mechanisms [20].

Patient data collection in healthcare systems requires extensive resources to collect, input, share and analyze information to be used for certain medical services. Traditionally, patient information management has been slow, error-prone and unable to provide true real-time accessibility. A solution to this problem is to introduce cloud and edge computing to the healthcare system, thereby transforming the system to smart healthcare. This requires sharing clinical diagnoses information and patient monitoring. Rolim et al. (2010) propose automating the process using sensors attached to medical devices [21]. The sensors are connected to external gateways through wireless networks, and the information is stored in the cloud and is accessible to all medical staff. This proposal ensures a cost-effective, reliable cloud computing system that is integrated with medical devices. Doukas et al. (2010) develop a mobile system that provides storage, updating and retrieval of electronic healthcare data through cloud computing. The application greatly improves the management of patient health records and images [22].

Fog computing is an extended version of the cloud computing paradigm that enables new applications and services based on particular characteristics, such as low latency, location awareness, mobility, strong presence of streaming and real-time applications. A study by Bonomi proved the importance of fog computing and proposed that it is the appropriate application for smart healthcare services in smart cities [23]. Fong and Chung presented in their work the mobile cloud computing for healthcare systems where biomedical signals from multiple locations are continuously collected [24]. Miranda et al. [25] propose a platform that allows the management of healthcare systems. This platform implemented using several technologies is tested for eight months and the outcome evaluations proved the advantage of deploying IoT in the healthcare sector.

A milestone in the improvement of any dynamic system is the capability to apply enhancements without interrupting system operations. Thus, smart healthcare relies on simulation to improve operations, particularly in EDs, and Petri nets have proven to be an efficient approach to optimize these types of systems [26]. Jansen and Reijers [27] proposed a redesign of a mental health care institute using colored Petri nets. As a result, higher performance was achieved, service and flow times were reduced and the system was more efficient overall.

As stated earlier, the main problem facing healthcare systems over the last decade is the overcrowding of emergency departments, and this means that resources and work flow must be optimized. Dotoli et al. [28] propose a Petri net workflow model to improve the structure and dynamics of an ED at the general hospital of Bari, Italy. The model defined complete patient flow management, and proposed an optimization solution with new resource dimensions to guarantee maximum patient flow. A study by Mahulea et al. [29] proves that synchronization and concurrency make Petri nets powerful tools for modeling and analyzing healthcare systems. They presented a methodology for patient flow, using Petri nets to assign resources based on the type of activity required. Another approach for healthcare using Petri nets was proposed by Augusto and Xie [30]. A new methodology called MedPRO was modeled and integrated with simulation to address healthcare problems.

Fanti et al. [31] proposed an alternative to overcome ED overcrowding, suggesting early discharge from the ED and introducing a home care option. They proposed an integrated system using Petri nets to monitor patients from home, and to ensure communication among families, doctors, nurses and emergency call centers. Since then, many other researchers have applied Petri net simulation in their work, to study system flow and control resource and service allocation [32].

As found in the literature, Petri net modeling techniques have enhanced healthcare business processes and workflows [33,34,35,36,37]. A new simulator was proposed as well by Davidrajuh et al. in order to reduce the size of Petri net models for complex systems [38]. In addition, the use of cloud computing to manage and process healthcare data and resources is of significant importance, and edge technology will be a prime enabler of smart healthcare for smart cities.

## 3. Proposed Framework

The provision of smart city mainly relies on the integration of all smart systems including smart healthcare. The successful deployment of smart healthcare services depends on cloud and edge computing. The workflow and resource pools of the proposed smart healthcare system are defined in the cloud, where the process is executed and resources are assigned. Every resource has its own edge node that reports the completion of an assigned task. Then, the resource is reassigned by the scheduling algorithm in the cloud. Research has shown that cloud computing is more reliable, efficient and faster than regular client-server computing [39,40,41,42]. Having workflows in the cloud ensures that the process is always sound, due to fault tolerance policies supported by the cloud [43].

**Figure 1:**
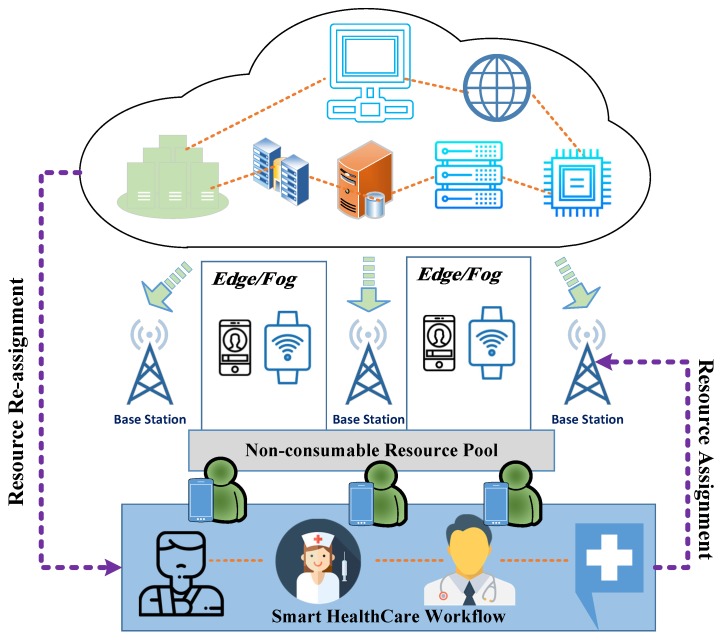
An edge based smart healthcare framework.

Figure 1 shows the high-level structure of a cloud-fog-based workflow system. The databases and workflow software are stored on the cloud, and task assignment and notification of completed processes are sent from the edge nodes to the cloud. Every resource has a smart device, such as a cell phone or tablet that works as an edge node. When the resource accomplishes the assigned task, it reports its status using the edge node to notify the cloud that the particular resource is available in the pool and ready to be re-assigned. The base station in Figure 1 shows that the communication mode among the players (Cloud, Edge, and medical team) is wireless and mobile.

In fact, we were referring to smart healthcare resource pool (i.e., Figure 1). We considered fog and edge are alike at this level. Both fog computing and edge computing are meant to push computing and processing capabilities closer to where the data originates. In our framework, the data is closer to the smart healthcare workflow.

As mentioned earlier, we broadly classify resources as consumable and non-consumable. A resource is considered non-consumable if it does not expire by time; otherwise, it is considered consumable. Human resources are an example of non-consumable resources, while consumable resources include items such as equipment, materials and any other non-human resources. This paper focuses only on non-consumable resources, and proposes a new Petri net called a Resource Preservation Net (RPN) that can be applied to any queuing system such as theaters, banks, etc. and including healthcare. A mathematical model to validate and better describe these systems is presented in Section 4. This RPN is an extended version of the general Petri net in the literature, with changes that allow a resource aware structure, and it is applied to a real-life problem presented in Section 8 to model and optimize an ED. A comparison of the general Petri net (PN) and the proposed RPN is presented in Figure 3.

**Figure 2:**
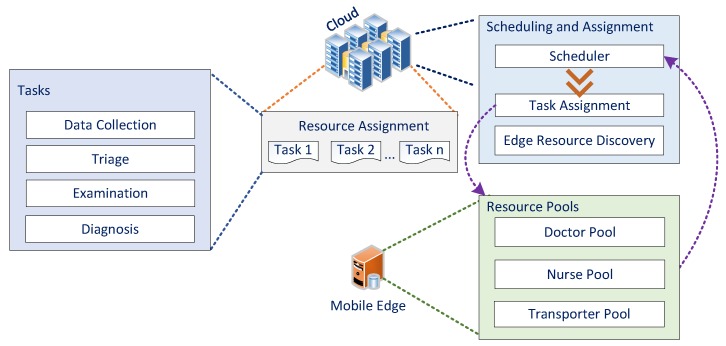
Overview of healthcare resource workflow.

Moreover, Figure 2 shows the two modes of the process: the cloud mode and the edge mode. Scheduling occurs in the cloud mode, and the assignment is sent to the edge. After the resource has completed the process, the edge mode notifies the cloud that the task is finished and it is ready for a new task.

**Figure 3:**
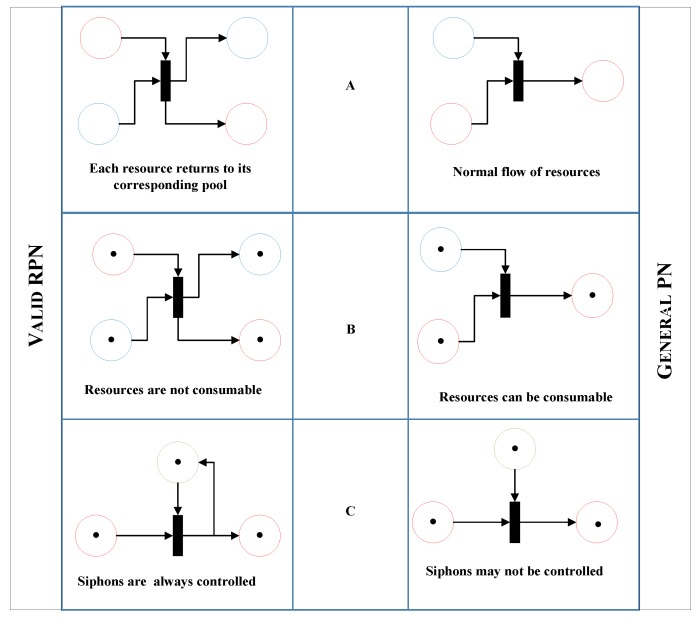
A comparison between RPN architecture (left-hand side) and regular Petri net (right-hand side).

In Figure 3A, as seen in the RPN (*left side*), for every place type in the set of input places of a certain transition, the same type exists as an output place. However, the number of input places and output places is not necessarily the same, as the place types must have a one-to-one relationship between input sets and output sets. Figure 3A, the general PN (*right side*) does not have this constraint, which means that certain types might be suppressed during transition execution.

Figure 3B shows the property of resource preservation. On the left, the RPN preserves the resource; that is, the number of input tokens is equal to the number of output tokens, while, on the right, tokens could be consumed as a general PN.

In Figure 3C, the left side shows the RPN with the property to control siphons, which is a basic property that must be satisfied in any RPN, and the right side of Figure 3C shows a general PN in which siphons do not need to be controlled.

## 4. Mathematical Model and System Validation

### 4.1. Preliminaries

Petri net is a well-known mathematical modeling technique that portrays the different states of a system. It is a directed graph built of two types of nodes: places, which are depicted as circles, and transitions depicted as rectangles. Places can be connected only to transitions, and transitions only to places. Petri net has proven to be efficient and reliable for describing and analyzing the flow of complex and concurrent systems, such as healthcare. Different types of nodes are connected using directed arcs, and places contain solid bullets which denote the tokens representing the activities performed by the transitions. A transition is fired or executed after it is enabled, and then tokens are removed from every input place of the fired transition and generated into each output place connected to that transition. Petri net has numerous features, including reachability and soundness.

**Figure 4:**
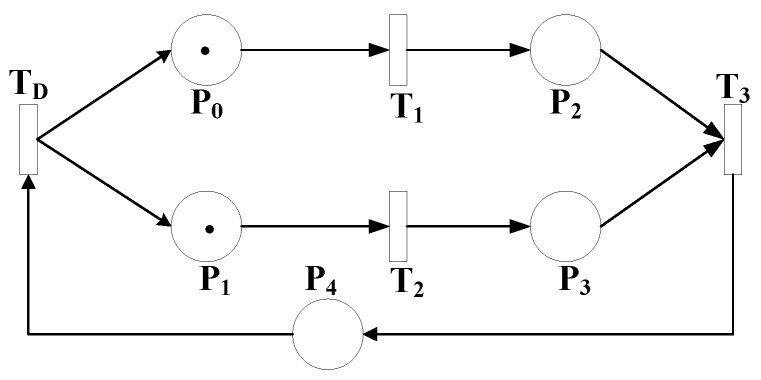
General Petri net model.

Reachability in this context is the ability to reach a certain node from another, and soundness is the ability to produce an output when given an input. Soundness is a very important Petri net feature because, if the net is sound, the topology of the workflow is correct. A general Petri net model is illustrated in Figure 4. The Petri net graph allows a model representation by using nodes that either represent transitions (added as rectangles) or places (added as circles). One transition can be connected to one or more places and vice versa, one place can be connected to one or more transitions. In case of identical types, the nodes will not be directly connected. Directed arcs are used to connect different types of nodes. Activities that are performed by the transitions are represented by tokens (solid circles) which reside in places. If a place (P) connected to a transition (T) is empty, the transition will not be executed. Therefore, an enabled transition exists if and only if no empty places are connected to this transition as input. The execution of a transition happens after it is enabled; we say the transition is fired. As a result of this firing, new tokens are created in each of the output places after removal of all tokens from each of the input places. The weights of arcs connecting the fired transition with its input and output places affect the number of tokens to be added or removed.

Many extensions and subclasses have been introduced in literature to solve concurrency problems. With Petri nets and workflows, tasks are modeled by transitions and dependencies by places and arcs [44]. When using Petri nets to model complex industrial workflows, systems are guaranteed to behave the way they are intended by two main Petri net characteristics: safety and soundness. Due to the complexity of healthcare systems and the many input factors, the systems can be seen as pipelines with stages that are considered critical sections; that is, the stages can serve only one patient at a time. Non-consumable resources are in pools, and, if they leave their pool to be involved in a task they need to return back to that pool after the task is completed, so they can be reassigned to another task. These critical sections must be handled with care, in order to maintain the soundness of the workflow system [45]. The importance of Petri net models as a detailed approach for formulating dynamic processes is presented by Huang et al. [46].

A Petri net ℵ is mathematically described as follows:

(1) ℵ=〈P,T,F,W〉,

where P is a non-empty set of places, T is a non-empty set of transitions, F is a non-empty set that represents the topology of the net, a set of arcs that joins places and transitions, and W which is the set of weights of those arcs. The set of arcs F is mathematically described as follows:

(2) F=(P×T)∪(P×T).

From Equation (2), it is obvious that places can only be connected to transitions and transitions can only be connected to places. No direct connection can exist between two places or two transitions. Every connection P×T or T×P has a weight which is an integer with a minimum value of one. This integer specifies the minimum number of activities required to flow at anytime through the arc.

Reachability is an important feature of Petri nets—that is, which node in the graph is reachable from which node. Figure 4 shows a Petrinet with full reachability because of the feedback between $T_{3}$ and $T_{0}$ through $P_{4}$. In this graph, we can say that $P_{2}$ is reachable from $T_{0}$ since there is a path that leads from $T_{0}$ to $P_{2}$. This is mathematically written as follows:

(3) $$P_{2}\in [T_{0}\rangle .$$

Tokens move inside Petri nets. They reside in places until being consumed by transitions during the firing process and then reproduced in other places according to the topology of the net. In Figure 4, $P_{0}$ has 1 token and $P_{1}$ has 1 token. This means that transitions $T_{1}$ and $T_{2}$ are enabled and $T_{3}$ and $T_{4}$ are disabled since the inputs of $T_{1}$ and $T_{2}$ have tokens and the inputs of $T_{3}$ and $T_{0}$ do not. Since $T_{1}$ and $T_{2}$ are enabled, they can fire and when they do, tokens are consumed from $P_{0}$ and $P_{1}$ (leaving them as empty places) and reproduced in $P_{2}$ ands $P_{3}$. Now, the only transition that is enabled is $T_{3}$ since all its inputs have tokens. When it fires, tokens are consumed from $P_{2}$ and $P_{3}$ and one token is reproduced in $P_{4}$, which, in turn, enables $T_{0}$. When $T_{0}$ fires, it consumes the token in $P_{4}$ and produces two tokens in $P_{0}$ and $P_{1}$.

The dynamic behavior of Petri nets is described by marking. Marking M is the distribution of tokens in the Petri net. It is represented as a vector with a length of ∥P∥. In other words, the length of the marking vector is as long as set P, where every place has a marking. The initial marking of the net is denoted $M_{0}$ and at time *t* to be $M_{t}$. The marking of place *j* at time *t* is denoted as $M_{t}\left(j\right)$.

The set of input places to a transition $T_{i}$ is denoted as $•T_{i}$ and the set of output places from a transition $T_{i}$ is denoted $T_{i}•$. The same goes for places. The set of input transitions to place $P_{j}$ is denoted as $•P_{j}$ and the set of output transitions from place $P_{j}$ is denoted as $P_{j}•$. In Figure 4, $\left\{P_{0},P_{1}\right\}\in T_{0}•$ and $\left\{P_{2},P_{3}\right\}\in •T_{3}$. Workflow nets are Petri nets with a single input place *i* and a single output place *o*.

The proposed Petri net model, RPN, is a workflow net that is validated using discrete mathematics. The validation is performed through proposing a Theorem of soundness. Mathematically, soundness is defined as follows:

(4) $$M_{t+\tau }\left(o\right)\in [M_{t}\left(i\right)\rangle ,\tau \geq 0,t\geq 0.$$

In other words, any input marking will eventually reach the output of the net.

### 4.2. System Validation

An RPN $\bar{ℵ}$ is a tuple that is defined as:

(5) $$\bar{ℵ}=\langle P_{p},ℵ,P_{p}\times ℵ\cup ℵ\times P_{p},R,\Theta \left(P_{p},R\right)\rangle$$

and

(6) ℵ=〈P,T,P×T∪T×P,W〉,

where $P_{p}$ is the set of pools of preserved resources, a set of controlled siphons. P is the set of places where $P\cap P_{p}=\emptyset$. T is the set of transitions. R is the set of resources, and $\Theta (P_{p},R)$ is the mapping function that assigns resources to pools.

A transition can be fired only if the required number of tokens at the input place is met. Different types of resources are defined in the system, each responsible for a certain task in order to accomplish the activity. We define the validity of a workflow to be the property of which the workflow behaves the way it is designed to behave. With this definition, we propose the following Theorem:

**Theorem** **1.** *A workflow $\bar{ℵ}$ is valid if*  *1*-
*ℵ is a sound workflow net.*
*2*-
$\forall r\in R,r\in M_{0}\left(P_{p}\right)$
*and*
$r\in M_{\infty }\left(P_{p}\right).$
*3*-
*If*
∃p∈P
*and*
$\exists P_{p}\in P_{p}∥P•\cap P_{p}•\neq \emptyset$
*then*
$M_{t}\left(P\right)\neq 0\to {\left(M\right)}_{t+\tau }\neq 0.$
*4*-$\forall P_{p}\in P_{p},P_{p}\times T\neq \emptyset ,T\times P_{p}\neq \emptyset$.*5*-∀T∈T, *if*$P_{p}\times T\neq \emptyset$*then*o∈[T〉.*6*-$\Sigma_{j=1}^{n}\left(M\left(P_{j}\right)\right)∥P_{j}\equiv •T_{s}=\Sigma_{k=1}^{m}\left(M\left(P_{k}\right)\right)∥P_{k}\equiv T_{s}•$.

Condition (1) of the Theorem states that the original workflow ℵ before applying the pools $P_{p}$ has to be sound. Condition (2) states that, for all the resources in the system, they have to exist in their pools at time 0 and after the process finishes execution (t=∞). Condition (3) states that, if there is a place *P* and a pool $P_{p}$ that are inputs to transition *T* and *P* is marked, then $P_{p}$ is guaranteed to be eventually marked. Condition (4) discusses the topological correctness of the workflow. It says that every pool has to be an input to a transition $T_{i}\in ℵ$ and an output from a transition $T_{j}\in ℵ$. Condition (5) guarantees that siphons will never affect the soundness of the original workflow ℵ. It states that, if there is a transition T∈ℵ with an input pool, then the output o∈ℵ is reachable from *T*. Condition (6) guarantees that no tokens are consumed inside any transition so the number of tokens used to enable a transition is the number of token that will be reproduced after it fires. We prove the Theorem as follows:

**Proof** **of** **Theorem** **1.** ∵ℵ is sound
∴
$\forall M_{t}\in [M_{0}\rangle$
(1)∵
$\forall P_{p}\in P_{p},\exists P_{p}\times T,\exists T\times P_{p}$

∵$\forall r\in R,r\in M_{0}\left(P_{p}\right)$ and $r\in M_{\infty }\left(P_{p}\right)$
∵$\forall P_{p}\in P_{p},P_{p}\times T\neq \emptyset ,T\times P_{p}\neq \emptyset$.
∵∀T∈T, if $P_{p}\times T\neq \emptyset$ then o∈[T〉
∴$P_{P}\times ℵ\cup ℵ\times P_{p}$ is a sound workflow(2) □∵
$\Sigma_{j=1}^{n}\left(M\left(P_{j}\right)\right)∥P_{j}\equiv •T_{s}=\Sigma_{k=1}^{m}\left(M\left(P_{k}\right)\right)∥P_{k}\equiv T_{s}•$

∴$\bar{ℵ}$ is sound(3)∴
$\forall m\in M_{t}\left(i\right),m\in M_{t+\tau }\left(o\right)$
(4)∴
$\forall r\in \bar{ℵ},r\in P_{p}$
(5)From (2), (3), (4) and (5)

$\bar{ℵ}$ is a valid RPN.

Theorem 1 defines the conditions that have to be applied for a workflow to be a valid RPN. Out of this theorem, we propose the following theorem:

**Theorem** **2.** *An RPN $\bar{ℵ}$ is sound if and only if $\bar{ℵ}$ is valid.*

To prove this theorem, we need to prove that if $\bar{ℵ}$ is valid then it is sound and if $\bar{ℵ}$ is sound then it is valid. To prove that if it is sound then it is valid is already done as a result of Theorem 1. The following is the proof that, if $\bar{ℵ}$ is valid, then it is sound:

**Proof** **of** **Theorem** **2.** ∵$\bar{ℵ}$ is valid
∴$\forall r\in R,r\in M_{0}\left(P_{p}\right)$ and $r\in M_{\infty }\left(P_{p}\right)$
∴$\forall P_{p}\in P_{p},P_{p}$ are well controlled siphons.
∵∀T∈T, if $P_{p}\times T\neq \emptyset$ then o∈[T〉□∴
$\forall m\in M_{t}\left(P\right),m\in M_{0}\left(i\right)$

∴
M(o)∈M(i)

∴$\bar{ℵ}$ is sound.

## 5. IoT and Healthcare

Smart healthcare depends on the cloud and edge computing. The workflow with its resource pools is defined on cloud where the process is executed and resources are assigned. Every resource has its own edge node where it reports the finishing of the assigned task. Afterwards, the resource gets reassigned by the scheduling algorithm in cloud.

The smart healthcare framework presented in this paper (Figure 1) along with healthcare resource workflow (Figure 2) is the reader guide to understand the relationship between the human factor and the framework. The human factor is referred to any kind of non-consumable healthcare service produced by the medical team and their stuff to share, allocate, or exchange medical information. The edge technology is the first backbone to store, process, and compute immediate data closer to the healthcare workflow while cloud is the second stage of data storage and processing like past patients’ histories. The fog/edge computing framework presented encompass the cloud, resources at the edge of the network, and healthcare system devices or sensors to execute certain related health tasks which can be seen from the communication flow modules in Figure 2.

This section is dedicated to validate the proposed Smart Healthcare (SHC) through a mathematical model:

(7) SHC=〈S,D,R,SR,DB,DA,E〉,

where SHC is a smart healthcare workflow, S is different workflow stages, D is a matrix that represents workflow dependencies, R is the set of workflow resources, SR is the stages’ resources, which is the cross product S×R. DB is a database to store history of events and all required transactions. DA is data accessibility, a security module that assigns data visibility to stages and finally E, the set of edge devices accessing data in an SHC system. In this section, we are using the same naming convention of the previous proposed workflow. In other words, $S_{j},S_{k}$ are different stages in S. $•S_{j}$ is a set of input places to stage $S_{j}$. $S_{j}•$ is the set of output places from stage $S_{j}$. S(i) is the first stage of the workflow. S(o) is the output stage of the workflow, and $S_{s}$ is a sequence of stages in the workflow.

The model proposed is valid if the following conditions are met:

1. ∀s∈S,∃r∈R|(r,s)∈SR. In other words, every stage requires some resources in order to accomplish required activities.
2. ∀r∈R,∃d∈DB|(r,d)∈DA. In other words, the resources involved in a certain activity should be able to access the database.
3. ∀e∃E,∃d∈DB|(d,e)∈DA. In other words, at each specific stage, even each resource should have an edge device which in turn has accessibility of data belonging to that specific stage.
4. $\forall S_{j},S_{k}\in S$, if $\exists (S_{j},S_{k})\in D$, then, $\exists e\in E|\left(S_{j},e\right)\Rightarrow S_{k}$. In other words, if two stages $S_{j}$ and $S_{j}$ are dependent, then one stage needs an edge device in order to reach another stage.
5. ∀r∈R,e∈E, if ∃(r,e), then r∈DA and e∈DA. In other words, if a resource has the edge device, he should have the right to access the data as well.

### Soundness of the SHC

A healthcare workflow is a workflow such that <S,R,e>. $S_{j}•\equiv •S_{k}\equiv e|e$ is a device that moves the resource from stage $S_{j}$ to stage $S_{k}$.

**Theorem** **3.** *A system SHC, $\bar{S_{HC}}$, is sound if and only if:* *1*-$\forall e\in E,\exists \mathrm{DA}|S_{j}•\neq \emptyset$*and*$•S_{k}\neq \emptyset$.*2*-$\forall S_{j},S_{k}\left|S_{j}\Rightarrow S_{k},\exists \in S_{j}\right|S_{j}\neq \emptyset$.*3*-$\exists S_{s}|S_{s}\in [S_{i}\rangle$*and*$S_{o}\in [S_{s}\rangle$.

Condition (1) states that, for each edge device belonging to a set of edge devices E, there exists data accessibility such that the output of a certain stage $S_{j}$ is never empty and the input of another stage $S_{k}$ is never empty. This means that there is a movement from one stage to another, which can be done only if there is data accessibility for the edge device. Condition (2) refers to the topology of the workflow. If the patient moves from one stage to another with the resource, this means that an edge device at each stage exists. Condition (3) states that there is a sequence of stages $S_{s}$ that exists, such that these stages belong to the reachability of the input stage $S_{i}$ and the output stage $S_{o}$ belongs to the reachability of the sequence of stages $S_{s}$. This means that patients are moving through the workflow from input to output and this can be possible in a smart healthcare workflow only if all the conditions listed here are met.

In other words, Theorem 3 guarantees that, for a sound Smart health care Workflow:

1. Each resource has an edge device.
2. Each resource has the privilege to access the database based on its role and the stage it is serving.
3. Each edge device can access the database pertaining to a certain stage.
4. Input is reaching output after a sequence of stages.

**Proof** **of** **Theorem** **3.** ∵$\exists S_{s}|S_{s}\in [S_{i}\rangle$ and $S_{o}\in [S_{s}\rangle$
∴
S(o)in[S(i)〉

∵$\exists e\in E|e\in S_{i}$ and since e∈DA
∴$\exists e•|e•=S_{i}•$,□∴∀d∈DB, if $d\in S_{i}$, then eventually, $d\in S_{o}$.
∴Therefore, the smart architecture, SHC, is sound.

## 6. RPN Application to Automation

**Figure 5:**
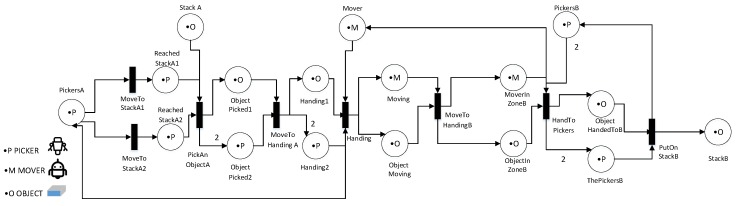
Two different types of mobile robots cooperate together to move a stack of objects from zone A to zone B.

While the focus of this paper is not robotics, this section is dedicated to applying the proposed model to robot automation in order to demonstrate the generality of the proposed framework. Figure 5 shows the environment and the setup of the proposed problem. It is an industrial environment where moving objects from zone A to zone B is required. Zone A has the objects stacked. It is required to move those objects into the stack in zone B. The assumption is that two types of heterogeneous robots are there. The first type robots are the pickers and the second type robots are the movers.

The process is assumed to be as follows:

1. Two pickers pick an object if available from stack A then hand it over to a mover.
2. The pickers go back to their home area (Pool) to be ready for another pick if availabe.
3. The mover moves the object from zone A to zone B.
4. The mover hands the object to two pickers in zone B.
5. The mover goes back to home zone (Pool) to move another object when ready.
6. The pickers of zone B pick the object and stack it on stack B.
7. The pickers go back to their home zone to be ready to pick another object from movers.

**Figure 6:**
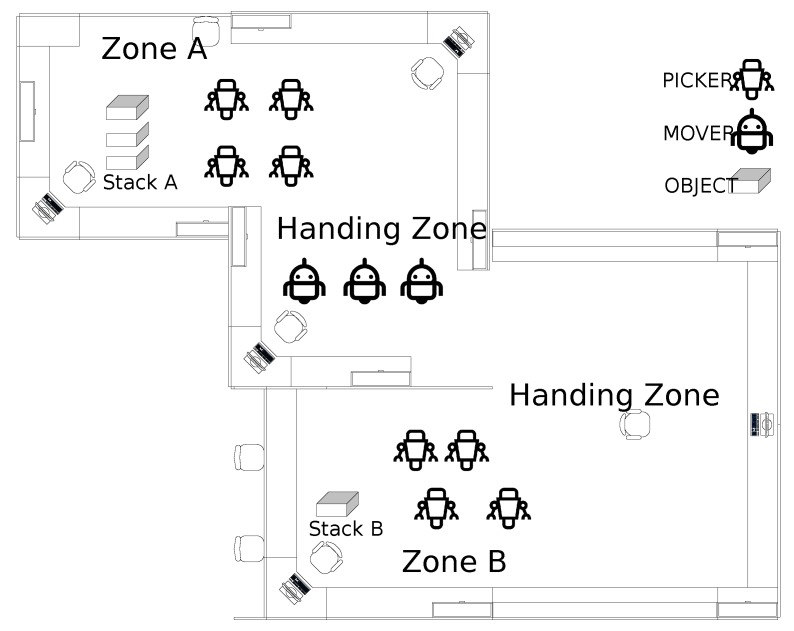
RPN structure of the robot automation process demonstrated in this section.

Figure 5 shows a proposed RPN structure for the problem described above. According to the mathematical description presented in Section 4, the problem is presented as follows:

- $P_{p}=\left\{P_{Mover},P_{Picker}\right\},$
- $P=\{Stack_{A},Stack_{B},ReachedStack_{A2},ReachedStack_{A1},ObjectPicked_{1},ObjectPicked_{2},Handing_{1},Handing_{2},Moving,ObjectMoving,MoverInZoneB,ObjectInZoneB,PickerB,ObjectHandedToB\},$
- $T=\{MoveToStackA_{1},MoveToStackA_{2},PickAnObject_{A},MoveToHanding_{A},Hand,MoveToHanding_{B},HandToPickers,PutOnStackB\},$
- $R=\{Picker_{1},Picker_{2},\cdots ,Picker_{8},Mover_{1},Mover_{2},Mover_{3}\},$
- Λ={Picking,Moving,Handing},
- λ is the request of pickers for movers or movers for pickers,
- F is the topology of the RPN shown in Figure 6,
- *i* is $Stack_{A}$,
- *o* is $Stack_{B}$,
- M=[10,4,0,0,0,0,0,0,3,0,0,0,0,4,0,0,0].
- $\Theta (P_{p},R)$ is the mapping function that assigns resources to pools.

λ here is a request that is being sent from movers to pickers or from pickers to movers. If the mover wants to hand objects to a picker, it sends it as a request λ and if the picker wants to take an object from a mover it sends λ.

For M, we assume that we start with $Stack_{A}$ as 10 objects. $Stack_{B}$ is initially empty. We have four Pickers in zone A, four pickers in zone B and we have three movers. M refers here to the initial marking of the Petri net which is defined in Section 4. The first value refers to the number of objects in the system which is 10. The second value refers to the number of pickers in Zone A which is 4. The third value refers to the number of movers which is 3 and the last value refers to the number of pickers in zone B which is 3. All other values are set to zero since places have no tokens and transitions did not fire yet. The robots are non-consumable resources that belong to their pools. The pools are controlled siphons by the definition of the RPN. The soundness of the model is given by the Theorems and their proves. Note that this paper is not forgetting the solution of cooperative robotics. Cooperative robotics set up is only to show the modelling capability of the proposed RPN. The robots are considered non-consumable resources as stated earlier.

## 7. RPN Application to Healthcare

**Figure 7:**
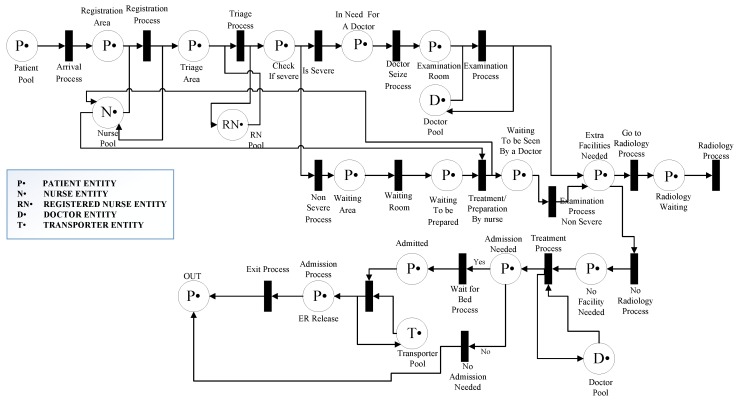
Emergency room stages.

**Figure 8:**
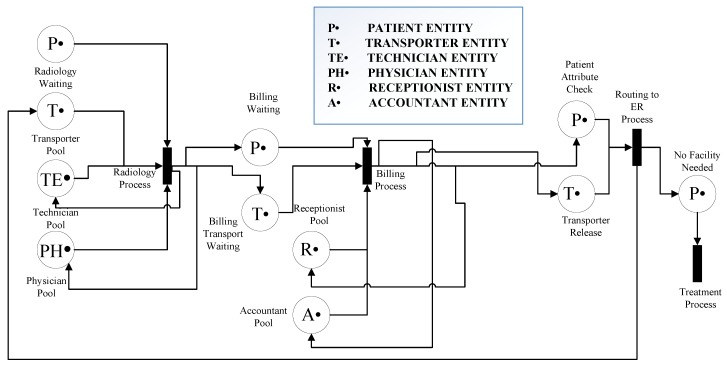
Petri net radiology/billing.

**Figure 9:**
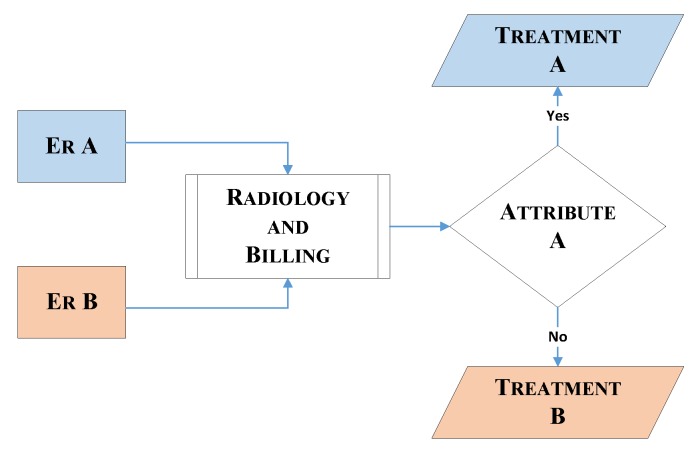
Petri net model flowchart.

The chosen ED operation and its flow of patients is shown using the proposed RPN. The PN framework is modeled by two RPNs, in order to discriminate between the two emergency rooms: *ER A* and *ER B*. The entities described in the model are the same for both ERs, and some, such as for radiology and billing, are common. The customer refers the patient and resources to the medical resources that serve the patient. In this RPN, the transitions represent each stage of the model, and the places represent the entity pools, such as patients or medical resources, and the transfer between stages. These places and transitions are connected with directed arcs, identified here as connections. Each entity in the model has a defined number of resources or tokens, which is known as marking. Figure 7 and Figure 8 represent the flow in each ER and the common units between the two ERs, respectively. The stages in Figure 7 are similar for both ER A and ER B, and the common units represented in Figure 8 are radiology and billing. Figure 7 describes the cooperation of the two ERs, and in Figure 9 patients leaving the radiology/billing stage move to the checking attribute stage. Patients will continue the flow by moving to Treatment A if they are from ER A, or Treatment B if they are from ER B. It is worth mentioning that, as shown in Figure 9, the patient, whether A or B, should follow the remaining flow after reaching the treatment stage.

The medical resource pools are known as the Doctor Pool, RN (Registered Nurse) Pool, Nurse Pool, Transporter Pool, Accountant Pool, Receptionist Pool, Physician Pool and Technician Pool. To control the critical sections in the model and avoid siphons, these resources should always return to their corresponding pools after accomplishing a particular task; we consider this an indication that the model is sound [47]. The only entity that flows from the beginning to the end of the system is the patient, which is not consumable. All the other resources are also non-consumable. The flow of patients in the system is from their arrival at the ER to when they leave, either to another unit (i.e., admitted) or home.

## 8. Simulation and Results

Simulation has proved to be an effective tool for evaluating and modeling processes over a certain period of time. Complex systems, such as smart healthcare, require simulation modeling to study the behavior of concurrent random elements, without the need to interrupt operations. Simulation modeling is a very useful method to solve system problems and test different scenarios, without the commitment or investment of physical resources.

A widely-used simulation tool that has been employed by many researchers to model concurrent, complex systems is the Arena Rockwell [48], which is a discrete-event simulation and automation software. The powerful Arena graphical templates assist module analysis, and help create wide, complex models. This tool is the basis of the simulation results obtained in this work. The first step in starting a simulation process is to formulate the problem and specify the objectives. For a successful simulation, it is essential to study and carefully plan three factors: people, cost and time. After studying the system, data collection should be performed to define dependencies among activities and probability distributions, as this system information is key to designing a model. At this stage, resources available in the real system must be involved in the data collection, since they are familiar with the actual flow of activities and processes. This ensures the accuracy and credibility of the model being designed. After construction and verification of the model, pilot runs are performed to test how it reacts to changes, and to adjust the input parameters in the case of undesired output. These pilot runs are an aspect of model validation, and the behavior of the model should be virtually the same as the real system under study. It is only after the simulation model is validated that experimentation can proceed. Experimentation is the process of defining the performance measures, and proposing optimization scenarios for system enhancement. As a 24/7 service, EDs will ultimately experience the problem of overcrowding, a growing issue largely due to the increase of different types of patients and different illness activities [49,50,51].

In this study, an ED is modeled and simulated using Arena. The ED consists of two emergency rooms (ER): ER A that offers public services, and ER B offering private services. Both ERs share same facilities, such as radiology and billing, and each has their own dedicated number of medical resources. Each has a waiting room for patients, a registration desk where patient information is collected, a doctor/nurse room where diagnosis tests are checked and decisions are made, a triage zone to classify the priority of arriving patients, three examination rooms, a surgical room where simple surgery can be done without admission and an ambulance arrival area from which high acuity patients are immediately transferred to an examination room. Patients arrive at the ED without prior notice and can follow different stages to receive the required care. The medical resources for these patients are included in the designed model and depicted in Table 1, where a waiting time affecting the overall process is generated.

**Figure 10:**
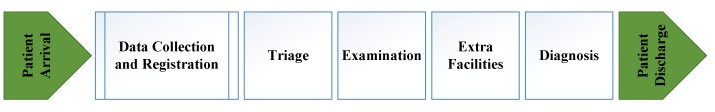
Patient journey in the ED.

The key stages followed by ED patients are: arrival, consultation, diagnosis, interpretation and the decision stage, which includes discharge or admission. This patient flow is illustrated in Figure 10. Upon arrival at the ED patient information, such as name, age, service needed and previous medical conditions, is collected by a nurse for a fee of $35, which is paid at the registration desk. The patient is then referred to a waiting room prior to the initial consultation. In the case of overcrowding in the ED, patients are referred to a registered triage nurse who decides which patient has the highest acuity level and needs attention first. The triage nurse is responsible for defining the acuity level of each patient, and referring critical cases to be addressed as soon as possible by an available doctor. The triage phase is followed by the ‘examination by a doctor’ stage, for which patients are transferred using a medical transporter or a nurse. Transporters are medical staff responsible for transferring patients from the ED to other units when extra facilities are required. Diagnosis begins when a doctor is available to consult with a patient in an examination room. Some patients are discharged with a prescription and medical recommendations if necessary, and others are advised to make use of facilities such as X-ray or CT scan. Once a patient has been transferred to the radiology unit and the imaging is done, they wait in a specified area for their image results and report. When the report is ready patients return to the ED with a transporter and wait again for the doctor to finalize the diagnosis. Before seeing the doctor again, the patient is requested to pay the imaging fee at the billing unit or process an insurance request.

In the final diagnosis step, patients could be discharged with prescriptions based on the imaging results or admitted to the hospital. In the case of admission, the patient will not be discharged from ED until a bed is available in an appropriate location. If simple surgery is recommended, it is performed in a dedicated area of the ED, which means that the patient must wait for an available specialized doctor and a registered nurse. A patient can only be discharged after the recovery time for their specific surgery has passed. Patients are served by the same doctor and nurses all along his journey in the ED. This “same patient-same staff” process has been shown to enhance system behavior. Finally, at the discharge stage, patients exit the system, which makes room for other patients.

Collecting arrival and service time at each stage helps to build a model that duplicates real system processes, where all data is fitted to corresponding distributions. This constitutes the simulation parameters. The simulated model is used to study the performance measures needed to improve the system. The model is run for 24 h, and the base time unit is always minutes. The performance measures recorded to validate the model are: the number of patients in and out (refer Table 2), patient LoS, service time spent in different queues, and the resource utilization rates. The number of patients reflects those who arrive at the ED, and the average number in this study was 138. The number of patients out reflects the number of patients exiting the system, and the average for this was 77 patients.

The length of stay (LoS) for the simulation output was 277 min for patient A and 294 for patient B, as shown in Table 3. LoS is a user-specified attribute that calculates the total time spent in the system from when a patient arrives until they leave. Resource utilization rates are presented in Table 4. The main issue is related to transporters and receptionists, where the average utilization rates are 97% and 93%, respectively. The average time patients spend waiting in queues is shown in Table 5.

**Figure 11:**
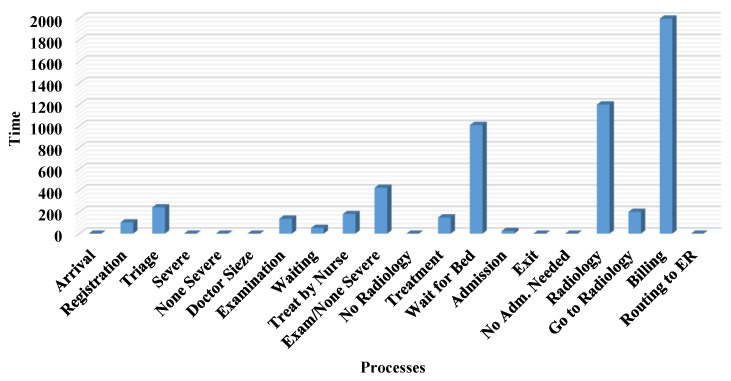
Units’ service time.

Figure 11 presents the simulation results of the proposed RPN model. They indicate that the billing unit suffers from bottlenecks, which is expected since the unit is shared by both ER A and ER B. Thus, the highest utilization rates were related to accountants and receptionist.

**Figure 12:**
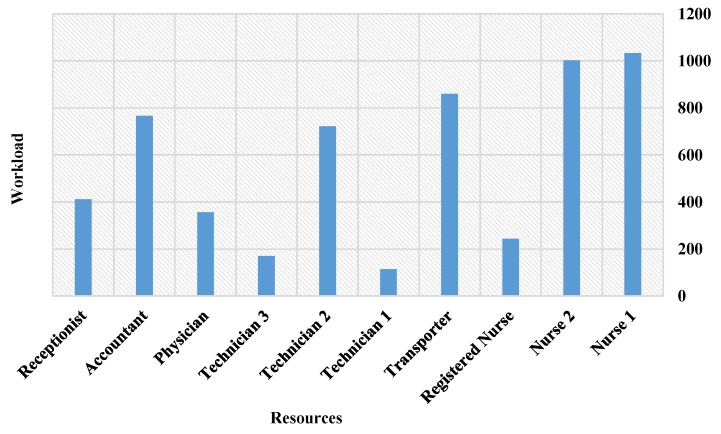
Resources workload.

As these resources are the busiest in the system, they should be considered for future optimization and, due to the simulation results, the radiology unit should also be considered. The workload of each resource is depicted in Figure 12. It is evident from the results that nurses are very busy and should also be considered for the future optimization, as well as technicians and accountants. Since the transporters wait for patients to complete an activity in another unit and then bring them from or to the ED, they also undergo high workload and lengthy service times. Future optimization should include more efficient allocation of these resources, and/or adding more medical resources. With new resource dimensions, average patient waiting times and resource usage could be decreased, while maintaining the same level of care. In previous work [52], ED simulation using Arena software (Arena 6th, Rockwell Automation, Coraopolis, PA, USA) was discussed in detail, and the results were similar to the RPN simulation outputs. This similarity in results validates the RPN simulation outputs, and means that the model is considered reliable and ready for future experimentation. Please state manufacturer, city and country from where equipment has been sourced.

## 9. Conclusions

Over the last decade, healthcare systems have been considered too complex to manage effectively, which has prompted decision makers to simply follow-up on daily operations and maintain a stable flow of patients. With IoT, smart cities and other new technologies, smart healthcare can solve the major ED problem of resource assignment efficiency. Due to reliability, cloud and edge computing are used to accommodate a proposed framework. This means that the soundness of the system will never be threatened, since cloud services do not malfunction and are automatically informed of required updates via edge devices. The RPN Petri net framework proposed in this paper will be useful for hospitals and other types of queuing systems. The framework considers only non-consumable resources, which always return to their pools after completing needed tasks, thereby ensuring control of system siphons. RPN is applied to one of two ERs in a general hospital, both of which operate separately and have their own medical resources, sharing only radiology and billing services. RPN describes the flow of patients in the ED from when they arrive until they are discharged, and it has proved to be sound through a new Theorem of soundness. RPN is an extended version of the general PN in the literature, with changes that allow a resource aware structure. As a summary of the new features of this extended PN, three factors must be highlighted: each resource returns to its corresponding pool, resources are not consumable, and siphons are always controlled.

Considering IoT to be the future of healthcare, many companies are investing in transforming their systems into smart healthcare systems where wearable devices are introduced for collecting data. Prompt solutions are also achieved by using Artificial Intelligence (AI) data assessment. The smart healthcare model defined in this paper helps in transforming the healthcare sector into a smart one by integrating cloud and edge computing. As for the future of smart healthcare, the help of robots may be approached for communicating, diagnosing and treating patients. As part of future work as well, RPN will be applied to other units of smart healthcare, and other theorems will be defined to study new operational flows and system soundness where cooperation between units will be introduced. Moreover, new privacy policies guaranteed by the framework will also be applied. Quality of Experience (QoE) of the provided services under the proposed smart healthcare framework will be also considered. As part of future work as well, the proposed concepts will be integrated as new hardware components in medical devices for a potential improvement in some healthcare services. Optimization will be integrated as well in order to study three satisfaction factors: customer, employee, and management.

## Figures and Tables

**Table 1:** Resources list and capacity in the model.

Resource Type	Role	Capacity
Doctor	Diagnosis and final decision	1
Nurse	Collecting patients information, preparing patients, providing care	2
Registered Nurse (RN)	Triage phase and head of nurses	1
Transporter	Transporting patients to other units	1
Technician	Available for extra facilities such as in the radiology unit	3
Physician	Available in Radiology unit to check the results and provide a report	1
Receptionist	Available for registration process and opening a file	1
Accountant	Responsible for billing	8
Specialist	A senior doctor	N/A

**Table 2:** Number of patients in/out.

Patients	Average	Half Width	Minimum Average	Maximum Average
**Number In**	138.00	2.655	128.00	140.00
**Number Out**	77.4000	4.501	71.0000	88.0000

**Table 3:** Patient LoS.

Tally Interval	Average	Half Width	Min Average	Max Average	Max Value	Max Value
Patient A	277.1100	60.071	163.140	401.750	16.2823	1050.08
Patient B	294.3200	45.244	183.670	347.510	16.0122	1029.51

**Table 4:** Resource utilization.

Resource Type	Utilization Percentage
Doctor A	35.28
Doctor B	35.59
Nurse A	26.57
Nurse B	26.74
Physician	39.86
Receptionist	93.32
Accountant	11.66
RN A	18.73
RN B	19.26
Technician	13.29
Transporter A	97.56
Transporter B	97.71

**Table 5:** Average time in queues.

Queue Type	Waiting Time (min)
Billing.Queue	9.7
Data Collection A.Queue	0.2656
Data Collection B.Queue	0.3834
Patient A Admitted to Hosp.Queue	28.4075
Patient B Admitted to Hosp.Queue	29.4428
Radiology.Queue	0.4442
Seize Doctor A.Queue	1.1220
Seize Doctor B.Queue	0.8771
Transporter A.Queue	410.31
Transporter B.Queue	426.31
Treatment A.Queue	1.9414
Treatment B.Queue	1.6706
Triage A.Queue	0.4498
Triage B.Queue	0.5023
Wait for bed A.Queue	0.05042
Wait for bed B.Queue	0.4665
Wait for Doctor A.Queue	1.5031
Wait for Doctor B.Queue	1.5245
Waiting Room A.Queue	0.0739
Waiting Room B.Queue	0.1142
